# Digital Twin Applications in Diabetes Management: Scoping Review

**DOI:** 10.2196/83059

**Published:** 2026-06-18

**Authors:** Fatemeh Sarani Rad, Maryam Jafarpour, Ehsan Bitaraf, Katayoon Khaleghdadi, Juan Li

**Affiliations:** 1 Computer Science Department, North Dakota State University, 1320 Albrecht Blvd, Fargo, ND, 58105, United States, 1 7012319662; 2 Institute of Outcomes Research, Center for Medical Data Science, Medical University of Vienna, Vienna, Austria; 3 Center for Technology and Innovation in Cardiovascular Informatics, Rajaie Cardiovascular Medical and Research Center, Iran University of Medical Sciences, Tehran, Iran; 4 Department of Computer Engineering, Islamic Azad University, Mashhad Branch, Mashhad, Iran

**Keywords:** digital twin, diabetes mellitus, continuous glucose monitoring, automated insulin delivery, machine learning, clinical decision support, ethics

## Abstract

**Background:**

Digital twin (DT) systems have emerged as a promising approach in health care, enabling real-time, patient-specific virtual modeling and personalized interventions. In diabetes care, DTs offer the potential to revolutionize glucose management, decision support, and therapy personalization through integration of real-time and longitudinal patient data.

**Objective:**

This scoping review mapped the current landscape of DT applications in diabetes and synthesized evidence across 13 research questions organized into 7 thematic domains: system design, target conditions, data sources, personalization strategies, intelligence and adaptability, validation methods, and implementation considerations.

**Methods:**

This scoping review was conducted in accordance with the PRISMA-ScR (Preferred Reporting Items for Systematic Reviews and Meta-Analyses extension for Scoping Reviews) and JBI methodological guidance for scoping reviews. A literature search was performed in PubMed, IEEE Xplore, Scopus, and Web of Science for studies published up to April 2025; all databases were last searched on June 23, 2025. Eligible studies were original empirical articles in English that described patient-specific DT systems or closely related individualized virtual models applied to diabetes diagnosis, monitoring, management, treatment, or complication-related care. Reviews, editorials, commentaries, theoretical papers without original data, and studies not focused on diabetes were excluded. Furthermore, FSR, MJ, and KK independently screened records and assessed full texts, with disagreements resolved through discussion and, when needed, by EB. Data were charted using a structured framework based on 13 predefined research questions, and were synthesized descriptively and thematically.

**Results:**

Of 208 records identified, 123 underwent title and abstract screening, 39 full texts were assessed for eligibility, and 28 studies were included. Most studies focused on type 1 or type 2 diabetes and used data-driven, hybrid, or simulation-based DT approaches. Common clinical applications included therapeutic control, glucose prediction, decision support, and disease management. Lifestyle data, wearables, continuous glucose monitoring, and electronic health records were the dominant inputs, while personalization relied on adaptive feedback, insulin optimization, and behavior-driven tools. Intelligent features, such as adaptive learning, explainable artificial intelligence, and real-time synchronization, enhanced adaptability, although human oversight was rare. Validation was mainly retrospective or simulation-based, with few clinical trials; reported outcomes included improved hemoglobin A_1c_, time-in-range, and reduced hypoglycemia. Ethical discussions focused on data privacy, while implementation barriers centered on validation gaps, data quality, and workflow integration.

**Conclusions:**

DT research in diabetes is expanding and shows strong potential for personalized and data-driven care; however, the evidence base remains heterogeneous, inconsistently reported, and limited in prospective clinical validation. Key gaps include standardized definitions, robust real-world evaluation, fairness and governance considerations, and integration into clinical workflows. Future work should prioritize clinically grounded validation, regulatory readiness, and interoperable architectures to support safe, equitable, and scalable implementation.

## Introduction

A digital twin (DT) is a dynamic, virtual representation of a physical system—such as a patient—that is continuously updated with real-world data and computational models to support prediction, simulation, and decision-making [1]. In health care, DTs are a powerful tool for personalized medicine, providing real-time, data-driven insights tailored to individual patients [2,3].

Diabetes mellitus, encompassing both type 1 and type 2 diabetes, remains a major chronic health condition requiring highly individualized care [4,5]. The complexity of diabetes management—driven by variability in disease trajectories, treatment responses, and complication risks—requires approaches that move beyond traditional one-size-fits-all models. DTs address this need by simulating glycemic dynamics, forecasting outcomes, and supporting therapy optimization on a patient-specific basis [2,3,6,7]. These models integrate diverse data sources, such as continuous glucose monitoring (CGM), insulin dosing records, electronic health records (EHRs), wearable sensors, genomic information, and lifestyle factors [3,6,7].

Recent research highlights the potential of DTs in diabetes for applications, such as predicting disease progression, personalizing nutrition, enhancing automated insulin delivery systems, and supporting self-management [3,7-11]. For instance, DT frameworks that combine machine learning, multimodal data, and mechanistic modeling have been used to predict glycemic and complication-related outcomes in diabetes [3,7,8,12,13]. Early-phase clinical and real-world studies suggest potential improvements in glycemic control, reduced medication use, and enhanced metabolic outcomes with DT-based interventions [8,11,14-18].

However, several barriers still hinder broader adoption and clinical integration. Key challenges include data integration and model personalization [3,6,7], limited interoperability across devices and systems [2,6,7], the absence of standardized validation and regulatory pathways [2,6,7], and unresolved concerns around data privacy and ethical use [2,6].

Despite promising progress, DT research in diabetes remains fragmented and undervalidated. While some reviews have examined digital health tools in diabetes or explored DTs in general health care contexts [6], no previous review has systematically synthesized DT applications in diabetes across key dimensions such as system design, personalization, data integration, validation, and implementation. This gap limits the ability of researchers, clinicians, and developers to assess maturity levels, identify best practices, and guide future development.

To address this gap, we conducted a scoping review guided by the following research questions. The review addresses 13 research questions (RQs) grouped under 7 thematic domains to improve clarity and synthesis.

1. System design and modeling foundations:
  - RQ1: What types of DT models have been developed for diabetes care and management?
  - RQ2: What system components are included in these models?
  - RQ3: What modeling approaches are used in these systems?
2. Target conditions and use context:
  - RQ4: What types of diabetes are addressed by these DT applications?
  - RQ5: What clinical goals do these DTs aim to support?
3. Data sources and personalization mechanisms:
  - RQ6: What data sources are used to build or update DTs for diabetes?
  - RQ7: How are DTs used to enable personalized care or self-management in diabetes?
4. Intelligence and adaptability:
  - RQ8: How do the DTs handle uncertainty, real-time data updates, and model interpretability?
5. Evaluation and validation:
  - RQ9: What outcomes have been reported from applying DTs in diabetes care?
  - RQ10: What methods have been used to validate these DT systems?
6. Implementation and governance:
  - RQ11: What ethical or legal issues are raised regarding the use of DTs in diabetes care?
  - RQ12: What barriers and enablers are reported for implementing DT systems in clinical practice?
7. Research and development gaps:
  - RQ13: What gaps in knowledge or practice are identified in the literature on DTs in diabetes?

By systematically synthesizing evidence across these domains, this review provides a comprehensive overview of the current state of DT research in diabetes. The findings aim to inform researchers, clinicians, and technology developers about prevailing trends, methodological practices, and future opportunities for advancing personalized diabetes care through DT technologies [2,4,5].

**Figure 1.**
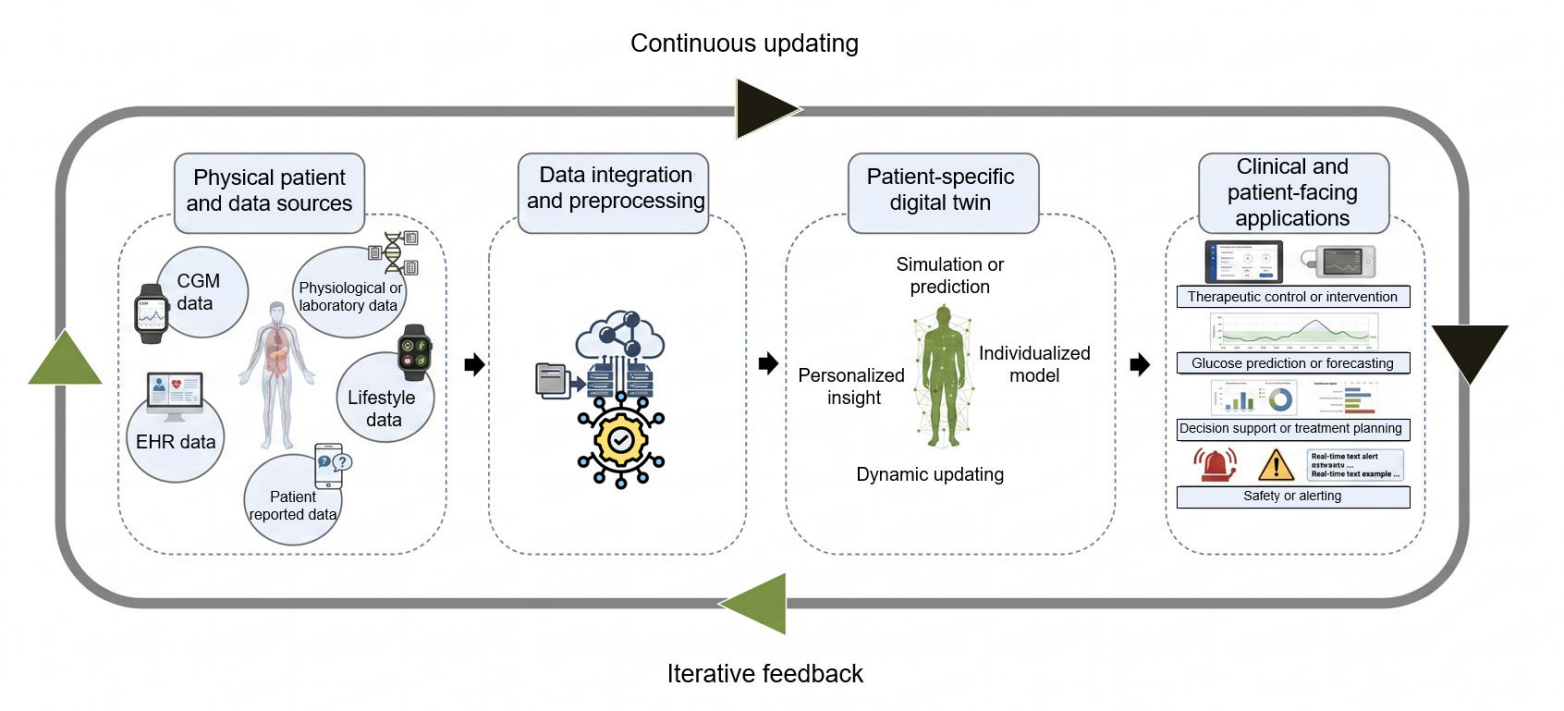
Synthesized architecture of digital twin systems in diabetes based on the included studies. Common components included multimodal patient data inputs, data integration and preprocessing, a patient-specific digital twin core, and clinical or patient-facing applications, such as therapeutic control, glucose prediction, decision support, treatment planning, and safety alerting. The outer loop represents continuous updating of the digital twin using incoming patient data and iterative feedback to support adaptive personalization. CGM: continuous glucose monitoring; EHR: electronic health record.

Figure 1 presents a synthesized architecture of DT systems in diabetes based on the common components identified across the included studies.

## Methods

### Overview

This scoping review was conducted in accordance with the PRISMA-ScR (Preferred Reporting Items for Systematic Reviews and Meta-Analyses extension for Scoping Reviews) and JBI methodological guidance for scoping reviews [19,20].

### Information Sources and Search Strategy

A comprehensive literature search was conducted through PubMed, IEEE Xplore, Scopus, and Web of Science. Studies published up to April 2025 were considered, and all databases were last searched on June 23, 2025. The search strategy combined terms related to “digital twin,” “diabetes,” and “healthcare” using Boolean operators. The detailed search strategy is provided in the Multimedia Appendix 1. Reference lists of included studies and relevant reviews were also manually screened to identify additional records.

The search strategies developed for PubMed, Web of Science, IEEE Xplore, and Scopus were imported into the Triple-A (Article Analysis Assistant) software [21]. The tool was used to integrate bibliographic metadata, automatically remove duplicate records based on DOI, and perform additional deduplication using title, publication year, and author names. Reviewer decisions were subsequently imported into the platform, and the finalized dataset was prepared for downstream analysis and thematic synthesis.

### Eligibility Criteria

Eligibility criteria were established a priori to ensure consistency and reproducibility during screening.

#### Inclusion Criteria

Studies were included if they were original empirical research articles, including peer-reviewed journal papers, conference proceedings, or preprints. Studies were eligible if they reported on the development, validation, implementation, or clinical evaluation of DT systems for diabetes, including type 1, type 2, gestational, or related complications. Research involving patient-specific modeling, simulation, or data-driven approaches relevant to diabetes diagnosis, management, or treatment was included. Articles addressing applications in personalized or precision medicine, clinical decision support, or individualized therapy for diabetes were also included. Publications were required to be written in English, with a structured abstract and an accessible full text.

#### Exclusion Criteria

Studies were excluded if they were review articles, meta-analyses, editorials, commentaries, book chapters, or theoretical or conceptual papers without original data. Studies focused on DTs for diseases or systems other than diabetes, such as cardiovascular, neurological, or orthopedic applications, were excluded. Articles lacking an abstract or full text, or published in languages other than English, were also excluded.

These criteria were set before the screening process to maintain consistency and transparency in study selection. During screening, FSR, MJ, and KK independently assessed each record for eligibility using the predefined criteria. Discrepancies or uncertainties were resolved through discussion, with EB consulted when necessary.

### Study Selection

The selection process involved 3 stages—identification, screening, and eligibility assessment. We initially identified 208 studies from 4 major databases—PubMed (n=47), IEEE Xplore (n=1), Scopus (n=107), and Web of Science (n=53). During identification, 85 articles were excluded due to duplication, lack of an abstract, absence of original data, or being published in a language other than English.

Following this step, 123 articles proceeded to screening. At the screening stage, 84 articles were excluded according to the predetermined exclusion criteria. As a result, 39 articles advanced to eligibility assessment, and 28 were included in the final review [3,7-18,22-36]. The 11 full-text articles excluded at the eligibility stage and the reasons for exclusion are listed in Multimedia Appendix 2.

Screening was conducted in 2 stages:

- Title and abstract screening: FSR, MJ, and KK independently assessed each record against the predefined eligibility criteria.
- Full-text screening: Articles passing the first stage were retrieved in full and assessed for final inclusion.

To ensure consistent inclusion decisions, the following screening questions were applied, reflecting the key characteristics of DTs and their application in diabetes care (Table 1). For the purposes of this review, a study was considered to describe a DT if it included a patient-specific virtual representation or individualized computational model linked to diabetes-related data and intended for prediction, simulation, monitoring, or decision support. Studies using terms such as “virtual patient” or “simulation model” were included only if these DT-defining characteristics were present. Generic population-level models without individualized representation or diabetes-specific application were excluded.

**Table 1.** Filtering questions for study selection.

Screening question	Decision criteria
FQ1^a^: Does the study discuss or apply DT^b^ technology?	Include only if the study explicitly referred to a DT or described a patient-specific virtual representation or individualized computational model linked to diabetes-related data and intended for prediction, simulation, monitoring, or decision support.
FQ2: Is the study focused on diabetes or diabetes-related conditions?	Include only if the main population or application domain involves diabetes (type 1, type 2, and gestational) or closely related metabolic conditions (eg, diabetic nephropathy and retinopathy).
FQ3: Is the DT model tailored to individual patients or based on patient-specific data?	Include only if the DT system is personalized using real or simulated patient-specific data (eg, glucose levels, insulin history, CGM^c^, and EHRs^d^). Exclude if the system is generic or population-level only.

a FQ: filtering question.

b DT: digital twin.

c CGM: continuous glucose monitoring.

d EHR: electronic health record.

### Data Extraction and Thematic Framework

Data from the 28 included studies were charted using a structured framework guided by the 13 predefined research questions introduced in the Introduction section. These research questions were organized into seven thematic domains to facilitate systematic synthesis: (1) system design and modeling foundations (RQ1, RQ2, and RQ3), (2) target conditions and use context (RQ4 and RQ5), (3) data sources and personalization mechanisms (RQ6 and RQ7), (4) intelligence and adaptability (RQ8), (5) evaluation and validation (RQ9 and RQ10), (6) implementation and governance (RQ11 and RQ12), and (7) research and development gaps (RQ13).

Each included study was analyzed systematically using this framework. Categories were not mutually exclusive, and individual studies could be charted under more than 1 category where appropriate. The full study characteristics and data charting table are provided in Multimedia Appendix 3.

Consistent with scoping review methodology, formal risk-of-bias, reporting bias, and certainty-of-evidence assessments were not performed because the aim was to map the breadth, characteristics, and gaps in a heterogeneous body of literature rather than to compare intervention effects or generate pooled estimates.

A review protocol and project materials for this scoping review were made available through the Open Science Framework (OSF) [37].

## Results

### Overview

**Figure 2.**
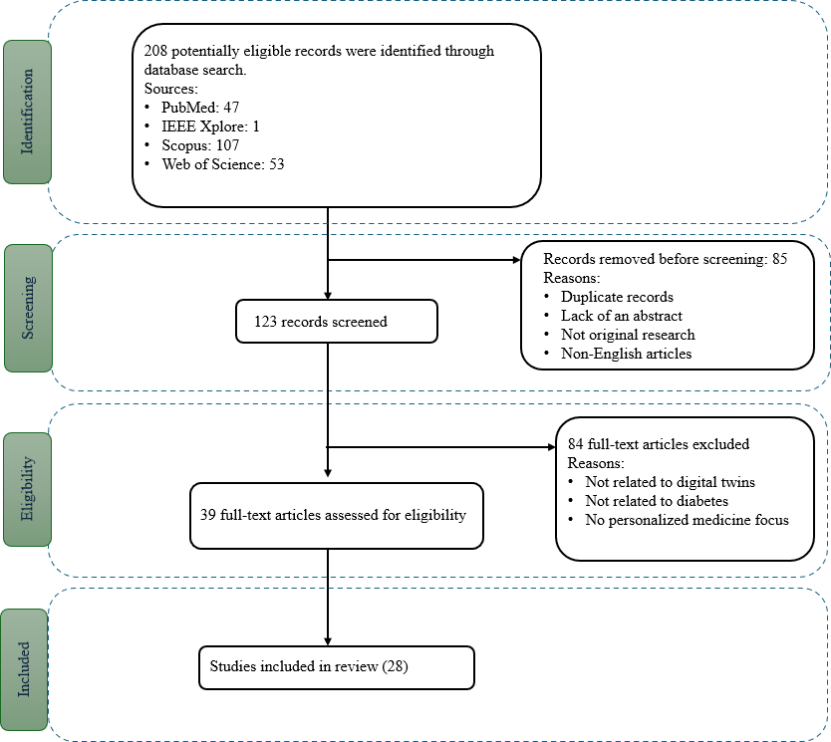
PRISMA (Preferred Reporting Items for Systematic reviews and Meta-Analyses) flow diagram of study selection process.

Across the 28 included studies (as shown in the PRISMA [Preferred Reporting Items for Systematic Reviews and Meta-Analyses] flow diagram in Figure 2) [3,7-18,22-36], DT systems for diabetes exhibited diverse architectures, data sources, and application goals. Most models were data-driven or hybrid (artificial intelligence [AI]+mechanistic), while purely mechanistic and conceptual designs were less common. Core system components included machine learning (ML) or AI modules, decision support layers, and real-time simulation engines. The majority of DTs leveraged CGM, wearables, and lifestyle data, with increasing use of patient-specific models to enable personalized therapy, behavioral nudges, and simulation-based feedback.

ML was the dominant modeling approach, while reinforcement learning, control theory, and signal processing appeared less frequently. Strategies for uncertainty management and interpretability were adopted inconsistently, with adaptive learning and explainable AI used in some studies, but with limited human-in-the-loop oversight. Reported outcomes most often focused on glycemic control (eg, hemoglobin A_1c_ [HbA_1c_], time-in-range [TIR], and reduced hypoglycemia), alongside improvements in predictive accuracy, metabolic markers, and patient engagement. However, external clinical validation remained scarce, with most evaluations based on retrospective datasets or simulations.

Ethical considerations—mainly privacy and transparency, with occasional references to accountability and bias—were inconsistently addressed. Implementation barriers included validation limitations, data quality issues, model limitations, and workflow misalignment. Finally, the literature highlights persistent research gaps in integration with real-world systems, scalability, and methodological rigor that must be addressed to advance DT systems into clinical use.

### System Design and Modeling Foundations (RQ1, RQ2, RQ3)

#### Overview

This section describes how DT models in diabetes are structured and modeled. It summarizes the types of models used (RQ1), the core system components included (RQ2), and the computational modeling strategies adopted (RQ3). Together, these questions cover the architectural and technical foundations of DTs in diabetes.

#### Model Types (RQ1)

DT models in diabetes care fall into 4 main categories—data-driven, hybrid, mechanistic, and conceptual. Data-driven models—most commonly using ML or deep learning—were used in half of the studies and focused on prediction and classification tasks. Hybrid models, which combine physiological modeling with AI, support real-time control systems, such as automated insulin delivery. Mechanistic models appeared less frequently and were primarily used in simulation studies. Conceptual frameworks were rare and largely theoretical. Table 2 summarizes the types of DT models reported in diabetes care, with representative examples from included studies.

**Table 2.** Types of digital twin models used in diabetes care with representative examples from included studies (research question 1). Percentages may exceed 100% because individual studies could be coded into more than one category.

Model type	Key characteristics	Studies, n	Representative examples
Data-driven	ML^a^, DL^b^, RL^c^; CGM^d^-based	14	Shamanna et al [8], Shamanna et al [14], Vaskovsky and Chvanova [22], Shamanna et al [15]
Hybrid	ML+mechanistic model	10	Sarani Rad et al [3], Cappon et al [9], Colmegna et al [23], Ahmadasas et al [10]
Mechanistic	ODEs^e^, simulations	5	Young et al [24], Thamotharan et al [25], Wang et al [26], Zavitsanou et al [27]
Conceptual	Framework only	1	Mishra et al [28]

a ML: machine learning.

b DL: deep learning.

c RL: reinforcement learning.

d CGM: continuous glucose monitoring.

e ODE: ordinary differential equation.

Key findings included:

- Data-driven models (14 studies, 50%): Applied for HbA_1c_ forecasting, glycemic risk scoring, and behavior modeling [8,11-18,22,31,32,34,35].
- Hybrid models (10 studies, 35.7%): Enabled adaptive insulin dosing and feedback control by integrating ML with mechanistic physiology [3,7-10,23,29,30,33,36].
- Mechanistic models (5 studies, 17.9%): Focused on ODE-based glucose-insulin dynamics for simulation and metabolic exploration [24-28].
- Conceptual frameworks (1 study, 3.6%): Proposed theoretical DT architecture without implementation [28].

#### System Components (RQ2)

Most DT systems consisted of modular components supporting prediction, simulation, control, and user interaction. The most common modules were ML or AI components, followed by simulation engines and data integration layers. User-facing dashboards and decision support or control modules were also frequently described, while personalization layers, backend infrastructure, and rule-based systems were less common. Table 3 summarizes the system component categories reported in diabetes DT models, including their functions and representative examples.

**Table 3.** System component categories in diabetes digital twin models with representative examples from included studies (research question 2). Percentages may exceed 100% because individual studies could be coded into more than one category.

System component category	Key characteristics	Studies, n	Representative examples
ML^a^/AI^b^ module	LSTM^c^, CNN^d^, reinforcement learning	20	Zhang et al [7], Pellizzari et al [29], Chen et al [30], Joshi et al [11]
Simulation engine	Glucose-insulin model, ReplayBG engine, ODE^e^-based simulator	17	Wang et al [26], Zavitsanou et al [27], Mishra et al [28], Leszczełowska et al [31]
Data integration layer	CGM^f^ devices, IoT^g^ sensors, preprocessing layer	12	Vaskovsky and Chvanova [22], Ahmadasas et al [10], Villa-Tamayo et al [32], Shamanna et al [16]
User interface or dashboard	Mobile apps, web dashboards, patient interfaces	11	Colmegna et al [23], Leszczełowska et al [31], Shamanna et al [17], Shamanna et al [18]
Decision support or control feedback module	MPC^h^, PID^i^ controller, feedback system	10	Shamanna et al [8], Cappon et al [9], Young et al [24], Zhu et al [33]
Intervention or recommendation engine	GPT^j^-based module, precision nutrition, lifestyle recommendations	9	Sarani Rad et al [3], Shamanna et al [15], Young et al [24], Shamanna et al [17]
Personalization layer	Personalization engine, patient-specific tuning	5	Cappon et al [9], Young et al [24], Pellizzari et al [29], Chen et al [30]
Monitoring and alerts	Real-time alerts, patient monitoring, CGM-based tracking	4	Shamanna et al [15], Shamanna et al [16], Shamanna et al [18], Vaskovsky et al [34]
Backend or platform infrastructure	Cloud platform, database engine, analytics engine	3	Vaskovsky et al [34], Chahal et al [35], Cappon et al [36]
Knowledge representation or semantic layer	Knowledge graphs, ontologies	2	Sarani Rad et al [3], Zhang et al [7]
Rule-based decision system	Expert system, rule tables	2	Shamanna et al [8], Zhu et al [33]

a ML: machine learning.

b AI: artificial intelligence.

c LSTM: long short-term memory.

d CNN: convolutional neural network.

e ODE: ordinary differential equation.

f CGM: continuous glucose monitoring.

g IoT: internet of things.

h MPC: model predictive control___.

i PID: proportional-integral-derivative___.

j GPT: generative pre-trained transformer.

Key findings included:

- ML or AI modules (20, 71.4% studies) were central to prediction, therapy optimization, and personalization [7-14,16-18,22,23,29-35].
- Simulation engines (17, 60.7% studies) provided physiological modeling and glucose-insulin dynamics for testing and validation [3,7,9,13,15,17,18,24-31,33,36].
- Data integration layers (12, 42.9% studies) supported real-time data collection from CGM, internet-of-things sensors, and preprocessing pipelines [3,8,10,13,14,16,22,23,32,34-36].
- User interfaces (11, 39.3% studies) enabled interaction for patients and clinicians through mobile apps or dashboards [8,13,14,16-18,23,25,31,32,36].

#### Modeling Approaches (RQ3)

The computational strategies used in diabetes DT systems reflect both the predictive and control needs of these models. While ML was the dominant method, several studies incorporated reinforcement learning, control theory, and signal processing for adaptive and real-time decision-making. Table 4 summarizes the range of modeling techniques reported across included studies, with representative examples.

**Table 4.** Modeling techniques and approaches used in diabetes digital twin systems, with representative examples from included studies (research question 3). Percentages may exceed 100% because individual studies could be coded into more than one category.

Modeling approach category	Key characteristics	Studies, n	Representative examples
Machine learning	Random forest, LSTM^a^, CNN^b^, gradient boosting	20	Vaskovsky and Chvanova et al [22], Shamanna et al [15], Colmegna et al [23], Batagov et al [12]
Statistical or probabilistic methods	Logistic regression, Bayesian inference, survival analysis	9	Shamanna et al [15], Leszczełowska et al [31], Zhu et al [33], Vaskovsky et al [34]
Physiological modeling	ODEs^c^, mechanistic models, compartmental models	8	Cappon et al [9], Ahmadasas et al [10], Young et al [24], Wang et al [26]
Control theory	MPC^d^, optimal control, PID^e^ controllers	3	Ahmadasas et al [10], Wang et al [26], Zavitsanou et al [27]
Reinforcement learning	DQN^f^, Soft Actor–Critic	2	Sarani Rad et al [3], Chen et al [30]
Control, estimation, or signal processing	Kalman filtering, signal estimation, signal processing algorithms	3	Ahmadasas et al [10], Zavitsanou et al [27], Vaskovsky et al [34]
Optimization or model calibration	Parameter estimation, parameter fitting algorithms	2	Ahmadasas et al [10], Thamotharan et al [25]
Rule-based systems	Dynamic risk thresholding, equation-based bolus calculation, rule-based reasoning	2	Pellizzari et al [29], Zhu et al [33]
Simulation-based modeling	Simulation training, Euler’s method	2	Mishra et al [28], Chen et al [30]
Natural language processing	GPT^g^-based natural language generation	1	Cappon et al [36]
System dynamics	Causal loop diagrams, feedback modeling	1	Mishra et al [28]

a LSTM: long short-term memory.

b CNN: convolutional neural network.

c ODE: ordinary differential equation.

d MPC: model predictive control ___.

e PID: proportional-integral-derivative__

f DQN: deep Q-network___.

g GPT: generative pre-trained transformer.

Key findings included:

- ML was the most common approach (20, 71.4% studies), used for glucose prediction, patient modeling, and feature extraction [3,7-9,11-18,22,23,27,31-35].
- Statistical and probabilistic methods appeared in 9 (32.1%) studies, often applied to regression, inference, or survival analysis [3,7,8,15,23,31,33,34,36].
- Physiological modeling was reported in 8 (28.6%) studies, leveraging ordinary differential equations, compartmental models, and mechanistic representations [9,10,24-26,29,30,36].
- Control-based approaches were less frequent, with control theory (3, 10.7% studies) [10,26,27] and reinforcement learning (2, 7.1% studies) supporting adaptive insulin delivery and personalization [3,30].

### Target Conditions and Use Context (RQ4, RQ5)

#### Overview

This section summarizes the specific types of diabetes addressed in DT studies (RQ4) and the clinical goals these models aim to support (RQ5). Together, these questions provide insight into intended use cases and patient populations for DT applications in diabetes care.

#### Target Conditions (RQ4)

DT studies in diabetes addressed multiple forms of the disease, with some models applicable to more than 1 type. Most studies focused on type 1 diabetes (T1D) or type 2 diabetes (T2D), whereas fewer studies targeted gestational diabetes or diabetes-related complications. Table 5 summarizes the targeted diabetes types and disease stages addressed across the included studies, with representative examples.

**Table 5.** Targeted diabetes types and disease stages addressed in included studies with representative examples (research question 4).

Diabetes type	Studies, n	Representative examples
Type 2 diabetes	14	Colmegna et al [23], Mishra et al [28], Villa-Tamayo et al [32], Shamanna et al [18]
Type 1 diabetes	13	Thamotharan et al [25], Wang et al [26], Zavitsanou et al [27], Batagov et al [12]
Diabetic retinopathy (secondary to diabetes)	1	Chahal et al [35]
Gestational diabetes	1	Leszczełowska et al [31]

Key findings included:

- T2D was the most frequent target (14, 50% studies), with models supporting therapy optimization, metabolic simulation, and lifestyle interventions [3,7-9,13-18,23,28,32,34].
- T1D was addressed in 13 (46.4%) studies, primarily through closed-loop systems, real-time insulin delivery, and glucose control simulations [3,10-12,22,24-27,29,30,33,36].
- Diabetic complications were rarely considered, with 1 (3.6%) study focused on diabetic retinopathy [35].
- Gestational diabetes was examined in 1 (3.6%) study, reflecting limited application to pregnancy-related diabetes [31].

#### Clinical Goals (RQ5)

DT applications in diabetes addressed a broad range of clinical objectives, spanning real-time monitoring, safety, decision support, and long-term disease management. These goals were classified into primary categories reflecting their roles in clinical care. Table 6 summarizes the clinical goals of diabetes DTs, including their functions and representative examples.

**Table 6.** Clinical applications of digital twin models for diabetes care with representative examples from included studies (research question 5). Percentages may exceed 100% because individual studies could be coded into more than one category.

Clinical goal category	Key characteristics	Studies, n	Representative examples
Therapeutic control or intervention	Insulin dosing, glycemic variability management, closed-loop control	17	Colmegna et al [23], Wang et al [26], Zavitsanou et al [27], Shamanna et al [18]
Decision support or treatment planning	Dietary recommendation, therapy optimization, clinician support	10	Colmegna et al [23], Thamotharan [25], Mishra et al [28], Cappon et al [36]
Safety or alerting system	Hypoglycemia alerts, early glycemic warnings, safety enhancement	10	Young et al [24], Pellizzari et al [29], Chen et al [30], Joshi et al [11]
Disease prediction or forecasting	Glucose forecasting, disease progression prediction, GDM^a^ risk	9	Sarani Rad et al [3], Shammana et al [8], Zhang et al [7], Joshi et al [11]
Disease management or remission	HbA_1c_^b^ reduction, weight loss, medication reduction	8	Shamanna et al [8], Shamanna et al [14], Shamanna et al [16], Surian et al [13]
Monitoring or control	Glucose time-in-range, health monitoring, normoalbuminuric	6	Sarani Rad et al [3], Young et al [24], Mishra et al [28], Shamanna et al [16]
Diagnosis or screening	DR^c^ detection, GDM diagnosis, complication screening	4	Zhang et al [7], Leszczełowska et al [31], Vaskovsky et al [34], Chahal et al [35]
Risk assessment	Maternal risk, risk stratification	3	Leszczełowska et al [31], Villa-Tamayo et al [32], Vaskovsky et al [34]

a GDM: gestational diabetes mellitus.

b HbA_1c_: hemoglobin A_1c_.

c DR: diabetic retinopathy.

Key findings included:

- Therapeutic control or intervention was the most common application (17, 60.7% studies), including insulin dosing, closed-loop control, and management of glycemic variability [3,8,10,13-18,23,25-27,29,30,33,36].
- Decision support and treatment planning were reported in 10 (35.7%) studies, covering dietary recommendations, therapy optimization, and clinician-facing guidance [3,7-9,23-25,28,33,36].
- Safety and alerting systems also appeared in 10 (35.7%) studies, emphasizing hypoglycemia warnings and proactive risk alerts [10-12,24-27,29,30,34].
- Disease prediction or forecasting was described in 9 (32.1%) studies, targeting HbA_1c_ trajectories, disease progression, and gestational diabetes risk [3,7-9,11,12,22,31,32].

In Table 6, “Therapeutic control or intervention” refers to systems that actively optimize or recommend treatment actions, such as insulin dosing or therapy adjustment; “monitoring or control” refers to systems focused on tracking glycemic status or physiological trends; and “decision support or treatment planning” refers to systems that inform clinician or patient decision-making without necessarily acting as real-time controllers.

### Data Sources and Personalization Mechanisms (RQ6, RQ7)

#### Overview

This section summarizes the types of data used to construct or update DTs for diabetes (RQ6) and the mechanisms through which these models enable personalization or self-management (RQ7). These aspects reflect both the technical input and patient-centered application of DT systems.

#### Data Sources (RQ6)

DT models drew on a wide range of data sources to ensure an accurate representation of patient state and dynamics. These included lifestyle, sensor-derived, clinical, and synthetic datasets, with varying degrees of adoption across studies. Table 7 summarizes the data sources used in diabetes DT systems, with representative examples.

**Table 7.** Clinical and behavioral data sources used in digital twin systems with representative examples (research question 6). Percentages may exceed 100% because individual studies could be coded into more than one category.

Data category	Key characteristics	Studies, n	Representative examples
Lifestyle data	Physical activity, dietary intake, sleep patterns	20	Colmegna et al [23], Zavitsanou et al [27] , Shamanna et al [17], Shamanna et al [18]
Wearable devices	Heart rate, insulin delivery data, blood pressure,	19	Sarani Rad et al [3], Chen et al [30], Shamanna et al [16], Shamanna et al [17]
CGM^a^	CGM data, blood glucose measurements, glucose monitors	18	Vaskovsky and Chvanova [22], Colmegna et al [23], Shamanna et al [17], Shamanna et al [18]
Electronic health records	Clinical history, laboratory results, patient demographics	12	Shamanna et al [15], Villa-Tamayo et al [32], Shamanna et al [17], Shamanna et al [18]
Simulated and public datasets	PIMA^b^ dataset, UVa/Padova simulator, synthetic NHANES^c^ data	6	Wang et al [26], Zavitsanou et al [27], Mishra et al [28], Chahal et al [35]
Physiological parameters	Body weight, personal characteristics, physiological metrics	5	Ahmadasas et al [10], Thamotharan et al [25], Zavitsanou et al [27], Pellizzari et al [29]
Patient-reported outcomes	Mobile health logs, self-monitoring, patient-generated input	3	Sarani Rad et al [3], Zhang et al [7], Pellizzari et al [29]
Genomic data	Metabolomics, proteomics	1	Zhang et al [7]
Imaging data	Fundus images, Optos scans, Gaussian-filtered visuals	1	Chahal et al [35]

a CGM: continuous glucose monitoring.

b PIMA: Pima Indians Diabetes Dataset.

c NHANES: National Health and Nutrition Examination Survey.

Key findings included:

- Lifestyle data were the most widely used input (20, 71.4% studies), covering physical activity, dietary intake, sleep, and behavioral logs [8-18,22-27,30,33,36].
- Wearable devices were incorporated in 19 (67.9%) studies, capturing heart rate, insulin delivery, and blood pressure [3,10-12,15-18,22-27,29,30,33,34,36].
- CGM appeared in 18 (64.3%) studies, enabling real-time tracking, control feedback, and risk forecasting [8,10-18,22-25,29,30,33,36].
- EHRs were used in 12 (42.9%) studies, providing longitudinal medical history, laboratory results, and medication data [3,7-9,13-18,32,33].

- Synthetic and public datasets were used in 6 (21.4%) studies, often for simulation or benchmarking, such as the UVa/Padova simulator or National Health and Nutrition Examination Survey (NHANES) data [26-28,31,32,35].

#### Personalization Mechanisms (RQ7)

Most DT systems aimed to enable personalized care through individualized feedback, adaptive modeling, or real-time decision support. Personalization strategies varied in scope, ranging from lifestyle guidance to therapy optimization and digital coaching. Table 8 summarizes the personalization features and tailoring strategies used in diabetes DT systems, with representative examples.

**Table 8.** Personalized features and patient-specific tailoring strategies in digital twin systems with representative examples (research question 7). Percentages may exceed 100% because individual studies could be coded into more than one category.

Personalization mechanism category	Key characteristics	Studies, n	Representative studies
Personalized lifestyle recommendations	Nutrition guidance, individualized meal or activity plans, lifestyle support	11	Cappon et al [9], Young et al [24], Shamanna et al [17], Shamanna et al [18]
Real-time or adaptive personalization	Dynamic feedback, CGM^a^-based tuning, adaptive intervention planning	11	Chen et al [30], Leszczełowska et al [31], Vaskovsky et al [34], Chahal et al [35]
Personalized insulin or therapy optimization	Personalized virtual patients, ReplayBG, health scenario simulation	10	Shamanna et al [15], Ahmadasas et al [10], Zhu et al [33], Cappon et al [36]
Self-management tools or patient interface	App feedback, color-coded food systems, personalized tracking tools	8	Sarani Rad et al [3], Shamanna et al [16], Shamanna et al [17], Surian et al [13]
Individualized simulation models	Personalized virtual patients, ReplayBG, health scenario simulation	6	Sarani Rad et al [3], Zavitsanou et al [27], Pellizzari et al [29], Chen et al [30]
Behavior-driven personalization	AI-guided nudges, digital coaching, human support	4	Shamanna et al [14], Colmegna et al [23], Shamanna et al [16], Surian et al [13]
Safety or alerting system	Tailored alerts, risk-specific notifications	1	Vaskovsky et al [34]

a CGM: continuous glucose monitoring.

Key findings:

- Personalized lifestyle recommendations were the most frequent approach (11, 39.3% studies), providing tailored nutrition, activity, and daily routine guidance [3,8,9,13-18,23,24].
- Real-time or adaptive personalization was also reported in 11 (39.3% studies), offering interventions dynamically responsive to CGM and sensor feedback [8,10,13,15,17,27,30,31,33-35].
- Personalized insulin or therapy optimization appeared in 10 (35.7% studies), focusing on precision dosing, medication planning, and adaptive therapy [3,9,10,15,18,23-25,33,36].
- Individualized simulation models were described in 6 (21.4%) studies, enabling patient-specific scenario testing and comparative evaluation [3,27,29-31,36].

### Intelligence and Adaptability (RQ8)

#### Overview

This section explores how DT systems in diabetes manage uncertainty, real-time data updates, and interpretability. These features are central to ensuring the trustworthiness, safety, and clinical relevance of DT models in dynamic health care settings.

#### Handling Uncertainty, Adaptation, and Interpretability (RQ8)

Based on the 28 included studies, 5 main categories of strategies were identified. Table 9 summarizes the strategies used for handling uncertainty, dynamic adaptation, and interpretability in diabetes DT systems.

**Table 9.** Reported strategies for managing uncertainty, real-time dynamics, and interpretability in diabetes digital twin models with representative examples (research question 8). Percentages may exceed 100% because individual studies could be coded into more than one category.

Strategy category	Key characteristics	Studies, n	Representative examples
Adaptive learning	Feedback loop tuning, model retraining, dynamic personalization	18	Shamanna et al [15], Thamotharan et al [25], Mishra et al [28], Vaskovsky et al [34]
Explainable AI^a^	Feature importance, knowledge graphs, visual interpretability	16	Vaskovsky and Chvanova [22], Colmegna et al [23], Wang et al [26], Chahal et al [35]
Real-time synchronization	Real-time CGM^b^ updates, Kalman filtering, continuous data sync	15	Colmegna et al [23], Ahmadasas et al [10], Wang et al [26], Chahal et al [35]
Confidence scoring	Cross-validation, confidence intervals, robustness testing	12	Vaskovsky and Chvanova [22], Zavitsanou et al [27], Mishra et al [28], Zhang et al [7]
Human-in-the-loop	Physician monitoring, manual oversight, feedback mechanisms	3	Shamanna et al [14], Mishra et al [28], Shamanna et al [18]

a AI: artificial intelligence.

b CGM: continuous glucose monitoring.

Key findings included:

- Adaptive learning was the most common capability (18, 64.3% studies), enabling dynamic personalization through feedback loop tuning, model retraining, and continuous parameter updates [3,8-10,13-18,23,25-28,30,33,34].
- Explainable AI appeared in 16 (57.1%) studies, using methods such as feature importance analysis, visual interpretability, and knowledge graphs to improve transparency [3,7,10-12,15,22,23,25,26,29,32-36].
- Real-time synchronization was reported in 15 (53.6%) studies, supporting continuous data integration from CGM and other sensors via Kalman filtering and real-time updates [3,8,10-14,16,18,23,26,27,33-35].
- Confidence scoring approaches were applied in 12 (42.9%) studies, employing cross-validation, CIs, and robustness testing to quantify uncertainty [7-9,11,12,15,22,27,28,30,32,36].

- Human-in-the-loop oversight was reported in 3 (10.7%) studies, providing physician monitoring or manual intervention in safety-critical contexts [14,18,28].

### Evaluation and Validation (RQ9, RQ10)

#### Overview

This section summarizes reported outcomes from DT applications in diabetes (RQ9) and describes the methods used to validate these systems (RQ10). Together, these questions address the effectiveness and credibility of DT models in clinical and experimental contexts.

#### Reported Outcomes (RQ9)

Across the 28 included studies [3,7-18,22-36], reported outcomes varied widely depending on the DT system’s clinical target and implementation maturity. Outcomes were grouped into major categories reflecting both clinical and system-level effects. Table 10 summarizes the clinical outcomes of DTs for diabetes.

**Table 10.** Clinical outcomes reported in digital twin research for diabetes, categorized by outcome type with representative examples (research question 9). Percentages may exceed 100% because individual studies could be coded into more than one category.

Outcome category	Key characteristics	Studies, n	Representative examples
Improved HbA_1c_^a^ or glycemic control	Increased time in range, HbA_1c_ reduction, improved control	17	Cappon et al [9], Thamotharan et al [25], Wang et al [26], Chen et al [30]
Other clinical benefits	Retinopathy or nephropathy improvement, cardiovascular risk reduction	11	Shamanna et al [8], Colmegna et al [23], Leszczełowska et al [31], Villa-Tamayo et al [32]
Improved prediction accuracy	Accurate glucose or GDM^b^ prediction, low RMSE^c^ or MAE^d^	9	Vaskovsky and Chvanova [22], Zavitsanou et al [27], Leszczełowska et al [31], Chahal et al [35]
Medication use reduction	Reduced or discontinued medication use	6	Shamanna et al [14], Shamanna et al [17], Shamanna et al [18], Surian et al [13]
Weight or metabolic outcomes	Weight loss, improved insulin resistance, BMI reduction	5	Shamanna et al [8], Shamanna et al [14], Shamanna et al [17], Surian et al [13]
Hypo- or hyperglycemia reduction	Fewer glycemic events, improved variability	5	Thamotharan et al [25], Zavitsanou et al [27], Chen et al [30], Zhu et al [33]
T2D^e^ remission or reversal	Diabetes remission or reversal	3	Shamanna et al [8]. Shamanna et al [15], Surian et al [13]
Improved detection or screening	Higher detection rates, classification accuracy	2	Mishra et al [28], Vaskovsky et al [34]
Blood pressure outcomes	Hypertension remission, reduced SBP^f^/DBP^g^	2	Shamanna et al [8], Shamanna et al [16]
Early detection or decision support	Improved early intervention	1	Vaskovsky et al [34]
Enhanced patient engagement	Improved patient comprehension and engagement	1	Sarani Rad et al [3]
Patient or clinician satisfaction	High clinician satisfaction	1	Zhu et al [33]
Personalized therapy optimization	Enhanced insulin dosing precision	1	Ahmadasas et al [10]

a HbA_1c_: hemoglobin A_1c_.

b GDM: gestational diabetes mellitus.

c RMSE: root mean square error.

d MAE: mean absolute error.

e T2D: type 2 diabetes.

f SBP: systolic blood pressure.

g DBP: diastolic blood pressure.

Key findings:

- Improved HbA_1c_ or glycemic control was the most frequently reported outcome (17, 60.7% studies), showing HbA_1c_ reduction, increased TIR, and reduced variability [3,8-10,13-15,17,18,23-27,29,30,33].
- Other clinical benefits were described in 11 (39.3%) studies, including retinopathy or nephropathy improvement and cardiovascular risk reduction [8,15-17,22,23,27,31,32,34,35].
- Improved prediction accuracy was reported in 9 (32.1%) studies, with accurate glucose or gestational diabetes mellitus prediction and low root-mean-square error (RMSE) and mean absolute error (MAE) [3,7,9,11,12,22,31,32,35].

- Less frequently, outcomes included medication use reduction, weight or metabolic improvements, hypo- or hyperglycemia reduction, and other patient-centered measures.

Reported quantitative outcomes suggest that some DT applications were associated with clinically meaningful improvements, although results varied by study design and use case. In 1 retrospective T2D cohort, HbA_1c_ decreased from 8.8% to 6.9% after 90 days, corresponding to a 1.9 percentage-point reduction, together with a 56.9% reduction in homeostatic model assessment of insulin resistance, a 6.1% decrease in body weight, and 89.1% (57/64) of participants achieving time in range (70‐180 mg/dL) ≥70% after the intervention [18]. In a DT-based exercise decision support system for T1D, mean time in range improved from 80.2% to 92.3% for aerobic exercise and from 72.3% to 87.3% for resistance exercise, while time spent in low glucose decreased from 15.1% to 5.1% and from 18.2% to 6.6%, respectively [24]. A mechanistic personalized nutrition model in prediabetes predicted individual body weight and HbA_1c_ trajectories with mean prediction errors of 0.7 kg and 0.08 percentage points in the training dataset, and approximately 1.1% and 1.4% percentage errors, respectively, in the test dataset [30]. Some prediction-focused systems also reported strong performance metrics, including RMSE 24.96 mg/dL, MAE 17.21 mg/dL, and area under the receiver operating characteristic curve >0.85 for postprandial glucose prediction, as well as area under the curve (AUC) of 0.80‐0.82 for chronic kidney disease identification and AUC 0.86 for 3-year chronic kidney disease prediction in T2D cohorts [8,13]. In maternal-risk applications, 1 DT system reported 83.5% accuracy for maternal health risk assessment and 97.2% precision for gestational diabetes prediction [31].

#### Validation Methods (RQ10)

Validation approaches were grouped into 5 broad categories, reflecting how DT systems were evaluated for performance, safety, and generalizability. Table 11 summarizes the validation methods used in diabetes DT systems.

**Table 11.** Validation methods used in diabetes digital twin studies with representative examples (research question 10). Percentages may exceed 100% because individual studies could be coded into more than one category.

Validation method category	Key characteristics	Studies, n	Representative examples
Quantitative evaluation	Accuracy metrics (eg, RMSE^a^ and AUC^b^), statistical tests, cross-validation	21	Vaskovsky and Chvanova [22], Mishra et al [28], Leszczełowska et al [31], Vaskovsky et al [34]
Retrospective validation	Cross-validation, train or test split, retrospective data analysis	10	Thamotharan et al [25], Joshi et al [11], Villa-Tamayo et al [32], Batagov et al [12]
Simulation testing	ReplayBG or UVa/Padova simulation, virtual cohort evaluation	9	Young et al [24], Wang et al [26], Pellizzari et al [29], Chen et al [30]
Clinical trials	Randomized controlled trial, pilot study, prospective design	4	Shammana et al [8], Shamanna et al [16], Zhu et al [33], Cappon et al [36]
Real-world validation	Clinical evaluation, patient outcomes, CGM^c^ tracking	4	Shamanna et al [8], Colmegna et al [23], Zhu et al [33], Surian et al [13]
Expert review	Case study evaluation, user feedback	2	Shamanna et al [23], Zhu et al [33]

a RMSE: root-mean-square error.

b AUC: area under the curve.

c CGM: continuous glucose monitoring.

Key findings included:

- Quantitative evaluation was the most common approach (21, 75% studies), typically using accuracy metrics (eg, RMSE, MAE, and AUC) and cross-validation methods to assess performance [7-9,11-18,22,24,27-32,34,35].
- Retrospective validation was applied in 10 (35.7%) studies, using historical datasets (eg, EHRs and CGM logs) for training or testing and retrospective analysis [8,9,11,12,22,25,31,32,34,35].
- Simulation testing was reported in 9 (32.1%) studies, often leveraging tools, such as the UVa/PADOVA simulator or ReplayBG, to validate insulin control and metabolic models [3,8,10,24-27,29,30].
- Clinical and real-world evaluation was limited, with clinical evaluation reported in 4 studies (14.3%) [8,16,33,36] and real-world evaluation reported in 4 (14.3%) studies [8,13,23,33], including small-scale pilots, randomized controlled trials, or deployment in real patient settings with CGM tracking.

- Expert review was rarely used, reported in 2 (7.1%) studies, based on clinician or user feedback or case study evaluation [23,33].

### Implementation and Governance (RQ11, RQ12)

#### Overview

This section describes how ethical, legal, and practical considerations are addressed in the implementation of DT systems for diabetes. It summarizes reported privacy and regulatory strategies (RQ11) and examines technical and workflow-related barriers to deployment (RQ12). Together, these questions assess readiness for safe, responsible, and scalable clinical integration.

#### Privacy, Ethical, and Regulatory Considerations (RQ11)

DT systems introduce complex ethical and legal considerations due to their reliance on sensitive health data and AI-driven decision-making. Among the 28 studies [3,7-18,22-36], 4 high-level categories were identified—data privacy, consent and transparency, accountability, and bias or fairness. Table 12 summarizes the strategies used for handling privacy, ethical, and regulatory issues in diabetes DT systems.

**Table 12.** Ethical, privacy, and regulatory considerations in diabetes digital twins with representative examples (research question 11). Percentages may exceed 100% because individual studies could be coded into more than one category.

Ethics or privacy category	Key characteristics	Studies, n	Representative examples
Data privacy	Data anonymization, encryption, GDPR^a^ or HIPAA^b^ compliance	8	Mishra et al [28], Zhu et al [33], Vaskovsky et al [34], Chahal et al [35]
Accountability	Audit trails, regulatory compliance, and interoperability	6	Cappon et al [9], Zhu et al [33], Vaskovsky et al [34], Chahal et al [35]
Consent and transparency	Data ownership, ethics approval obtained, informed consent, patient consent, permission-based data storage	6	Zhu et al [33], Vaskovsky et al [34], Chahal et al [35], Cappon et al [36]
Bias and fairness	Identification of bias potential	1	Sarani Rad et al [3]

a GDPR: General Data Protection Regulation.

b HIPAA: Health Insurance Portability and Accountability Act.

Key findings included:

- Data privacy was the most frequently discussed (8, 28.6% studies), typically through anonymization, encryption, and compliance with HIPAA (Health Insurance Portability and Accountability Act) or GDPR (General Data Protection Regulation) [3,8,9,28,33-36].
- Accountability appeared in 6 (21.4%) studies, including the use of audit trails, traceability, and regulatory compliance mechanisms [7-9,33-35].
- Consent and transparency were also reported in 6 (21.4%) studies, covering informed consent procedures, institutional review board approvals, and patient-facing disclosures [7,8,33-36].

- Bias and fairness were noted in only 1 (3.6%) study, reflecting a critical underexplored gap in addressing algorithmic inequity [3].

#### Implementation Barriers and Enablers (RQ12)

Although many DT systems demonstrated technical feasibility, real-world implementation remains constrained by several recurring challenges. These were grouped into 4 main categories—data quality and availability, model limitations, validation limitations, and workflow or interoperability barriers. Table 13 summarizes the implementation barriers that exist in diabetes DT systems.

**Table 13.** Implementation barriers and enablers in diabetes digital twin systems, with representative examples (research question 12). Percentages may exceed 100% because individual studies could be coded into more than one category.

Implementation barriers category	Key characteristics	Studies, n	Representative examples
Validation limitation	Lack of randomization, short follow-up, and personalization gaps	16	Wang et al [26], Zavitsanou et al [27], Villa-Tamayo [32], Shamanna et al [18]
Data quality or availability	Burden of data collection, missing variables, limited real-world data, and synthetic datasets	14	Valovsky and Chvanova [22], Wang et al [26], Shamanna et al [17], Shamanna et al [18]
Model limitations	Simplified physiology, tuning complexity, and selection bias	11	Ahmadasas et al [10], Wang et al [26], Leszczełowska et al [31], Villa-Tamayo et al [32]
Workflow and interoperability	Clinical workflow alignment, data format compatibility issues, data integration challenges, data integration from multiple sources, integration with existing devices, and interoperability challenges	8	Cappon et al [9], Colmegna et al [23], Mishra et al [28], Zhu et al [33]

Key findings included:

- Validation limitations were the most common barrier (16, 57.1% studies), reflecting reliance on synthetic datasets, short follow-up durations, and lack of external clinical evaluation [8,10-18,25-27,30,32,33].
- Data quality and availability issues were reported in 14 (50%) studies, including missing data, unreliable sensors, and burdensome data collection procedures [7,11,15,17,18,22,25,26,29-32,35,36].
- Model limitations were described in 11 (39.3%) studies, such as limited personalization, oversimplified physiological modeling, or small training datasets [3,7,10,13,14,16,26,27,29,31,32].

- Workflow and interoperability barriers appeared in 8 (28.6%) studies, emphasizing difficulties integrating DTs into clinical workflows, EHR systems, and device ecosystems [9,10,23,28,33-36].

### Research and Development Gaps (RQ13)

Although DT systems for diabetes are showing technical feasibility, multiple areas require further investigation and refinement. From the 28 reviewed studies [3,7-18,22-36], seven major gap categories that emerged were (1) limited scope of application, (2) integration challenges, (3) lack of longitudinal data, (4) data quality and availability, (5) methodological limitations, (6) need for clinical validation, and (7) scalability or usability concerns. Table 14 summarizes the reported research and development gaps in diabetes DT systems.

**Table 14.** Reported research gaps and future development needs in diabetes digital twin literature with representative examples (research question 13). Percentages may exceed 100% because individual studies could be coded into more than one category.

Gap category	Key characteristics	Studies, n	Representative examples
Need for clinical validation	Larger clinical trials and subgroup and demographic validation	15	Shamanna et al [15], Thamotharan et al [25], Shamanna et al [18], Zhu et al [33]
Limited scope of application	Broader populations, diverse settings, and multimorbidity expansion	14	Sarani Rad et al [3], Shamanna et al [15], Cappon et al [9], Thamotharan et al [25], Zhang et al [7]
Integration challenges	Integration with EHRs^a^, real-time systems, and closed-loop models	11	Thamotharan et al [25], Joshi et al [11], Zhu et al [33], Batagov et al [12]
Usability and real-world adoption	Personalization for MDI^b^ users, real-world evaluation, and broader adoption	11	Ahmadasas et al [10], Wang et al [26], Vaskovsky et al [34], Chahal et al [35]
Lack of longitudinal data	Long-term outcome tracking, sustainability, and effectiveness studies	8	Shamanna et al [14], Shamanna et al [15], Cappon et al [9], Surian et al [13]
Data quality and availability	Dependence on wearable devices and data quality, expansion to broader population data, expansion to larger datasets, limitations in meal tracking and calibration, and need for denser time-series data	6	Vaskovsky and Chvanova [22], Wang et al [26], Mishra et al [28], Villa-Tamayo [32]
Methodological limitations	Standardized protocols, adaptive learning, and causal reasoning	5	Sarani Rad et al [3], Vaskovsky and Chvanova [22], Colmegna et al [23], Pellizzari et al [29]
Scalability challenges	Deployment in low-resource settings and real-world scalability	3	Leszczełowska et al [31], Zhu et al [33], Chahal et al [35]

a EHR: electronic health record.

b MDI: multiple daily injection.

Key findings included:

- Need for clinical validation was the most frequently cited gap (15, 53.6% studies), reflecting the lack of randomized trials, subgroup evaluations, and real-world testing [3,9,13-16,18,23,25,27,29,31,33,34,36].
- Limited scope of application was reported in 14 (50%) studies, with DTs often targeting narrow use cases and failing to generalize across diverse populations or multimorbidity contexts [3,7-10,13,15-17,25-28,31].
- Integration challenges were noted in 11 (39.3%) studies, underscoring difficulties with EHR interoperability, real-time deployment, and multidevice environments [7,11,12,22,25,27,30,32-35].
- Usability and real-world adoption also appeared in 11 (39.3%) studies, pointing to the need for personalization, support for multiple daily injection users, and strategies for broader adoption in routine care [8,10,12,16,24,26,30,32,34-36].

## Discussion

This PRISMA-ScR–compliant scoping review maps the current state of DT systems in diabetes, addressing 13 structured research questions across 7 thematic domains.

### System Design and Modeling Foundations (RQ1, RQ2, RQ3)

DT systems for diabetes use a wide range of modeling techniques, most commonly ML (eg, long short-term memory, gradient boosting, and reinforcement learning) and physiological simulation. Simulation engines and predictive ML modules were often integrated into layered architectures that also included personalization modules, decision support, and user-facing dashboards. Statistical and probabilistic methods (eg, regression and Bayesian inference) were also used in several studies, although less prominently. Few systems incorporated mechanistic control theory or signal-processing models. The inclusion of key components, such as simulation engines, control-feedback modules, and data integration pipelines, reflects a growing maturity in system design.

### Target Conditions and Use Context (RQ4 and RQ5)

Most DTs targeted T1D or T2D, with limited applications in gestational diabetes or diabetes-related complications, such as retinopathy. Primary clinical goals included glycemic prediction, insulin-dose optimization, lifestyle guidance, and therapeutic planning. Several systems also addressed the diagnosis of complications or risk stratification for comorbidities. The breadth of clinical use cases suggests that DTs are evolving from simple simulators into multifunctional clinical-support tools.

### Data Sources and Personalization Mechanisms (RQ6 and RQ7)

Lifestyle data, wearable devices, and CGM were the dominant inputs, with hybrid combinations being common. EHRs and synthetic datasets were also widely used to provide historical or simulated information. Personalization was achieved through mechanisms such as real-time adaptation, individual model tuning, behavior-driven feedback (eg, nudges), and insulin titration. However, persistent challenges remain in data quality, sensor integration, and dataset heterogeneity.

### Intelligence and Adaptability (RQ8)

Managing uncertainty and real-time updates is crucial for clinical reliability. Studies implemented adaptive learning, feedback loops, and explainable-AI methods (eg, attention mechanisms and knowledge graphs) to improve transparency and adaptability. Real-time CGM synchronization and, in some cases, human-in-the-loop oversight were used to enhance model responsiveness and safety.

### Evaluation and Validation (RQ9 and RQ10)

Quantitative validation (eg, RMSE and AUC) was common, but real-world clinical trials were rare. Most studies validated systems via retrospective datasets or simulations. Reported clinical outcomes included improved TIR, fewer hypoglycemic events, and, in some cases, T2D remission. However, evidence on long-term effectiveness, generalizability, and cost-effectiveness remains limited.

A notable finding across the included studies is the mismatch between technical sophistication and clinical maturity. Although many DT systems incorporated adaptive learning, individualized simulation, and multimodal data integration, most were evaluated using retrospective datasets or in silico simulations rather than prospective clinical deployment. This likely reflects the high implementation burden of DTs in diabetes, including the need for reliable real-time data streams, safety safeguards, interoperability with devices and clinical systems, and acceptable workflow integration. It also reflects the regulatory complexity of systems that may influence insulin dosing or therapeutic decision-making.

### Implementation and Governance (RQ11, RQ12)

Privacy and ethical considerations were addressed inconsistently, often limited to brief compliance mentions (eg, GDPR and HIPAA). A smaller subset of studies explicitly discussed accountability (eg, audit trails and governance mechanisms) or algorithmic bias and fairness, highlighting underexplored areas of governance. Implementation enablers included real-time feedback and sensor integration, whereas barriers included poor data quality, system complexity, lack of clinical workflow alignment, and limited scalability.

Another important finding is the limited attention to algorithmic bias and fairness. Despite the increasing use of AI-driven modeling and decision-support approaches, only a small subset of studies explicitly discussed bias, representativeness, or equity-related concerns. This suggests that the field is still focused primarily on technical feasibility and predictive performance rather than equitable deployment across diverse patient populations.

### Research and Development Gaps (RQ13)

Key gaps include limited clinical validation, insufficient longitudinal data, a lack of standardized model architectures, and limited generalizability to diverse populations. Many studies emphasized the need for integration with EHRs, real-world testing, and regulatory alignment. Addressing these gaps will be essential to enable scalable, equitable, and clinically robust DT systems for diabetes management.

### Summary and Implications

This review offers a panoramic view of the evolving DT landscape in diabetes. While notable technical advances are evident—particularly in data integration and personalization—the field remains formative, with substantial work needed in clinical validation, ethical governance, and system interoperability. Future research should emphasize not only algorithmic sophistication but also real-world applicability, safety, and equity to support the scalable and responsible deployment of DTs in diabetes care.

Taken together, the literature suggests that DT research in diabetes is progressing from conceptual and simulation-based work toward more clinically relevant systems, but the field remains early in real-world maturity. Future studies should prioritize prospective validation, broader demographic and clinical representation, transparent reporting, interoperability with routine care systems, and governance frameworks that address privacy, accountability, and fairness.

### Limitations

This scoping review has several limitations. First, only English-language studies with accessible full text were included, and gray literature was excluded, which may have led to the omission of some relevant studies. Second, formal risk-of-bias and certainty-of-evidence assessments were not performed because the aim was to map a heterogeneous body of literature rather than evaluate intervention effects. Third, the included studies differed substantially in design, terminology, validation methods, and outcomes, limiting direct comparison. Finally, many studies were early-phase, retrospective, or simulation-based, which limits conclusions about clinical effectiveness and real-world implementation.

## Supplementary material

10.2196/83059 Multimedia Appendix 1 Search strategy.

10.2196/83059 Multimedia Appendix 2 Excluded full-text articles and reasons for exclusion.

10.2196/83059 Multimedia Appendix 3 Study characteristics and data-charting form.

10.2196/83059 Checklist 1 PRISMA checklist.

## Abbreviations

AI: artificial intelligence
AUC: area under the curve
CGM: continuous glucose monitoring
DT: digital twin
EHR: electronic health record
GDPR: General Data Protection Regulation
HbA_1c_: hemoglobin A_1c_
HIPAA: Health Insurance Portability and Accountability Act
MAE: mean absolute error
ML: machine learning
NHANES: National Health and Nutrition Examination Survey
OSF: Open Science Framework
PRISMA: Preferred Reporting Items for Systematic Reviews and Meta-Analyses
PRISMA-ScR: Preferred Reporting Items for Systematic Reviews and Meta-Analyses extension for Scoping Reviews
RMSE: root-mean-square error
RQ: research question
T1D: type 1 diabetes
T2D: type 2 diabetes
TIR: time-in-range
