# Supplementary material for: Digital Twin Applications in Diabetes Management: Scoping Review
Source: JMIR Diabetes. 2026 Jun 18;11:e83059. doi: 10.2196/83059 (PMC13277824; doi:10.2196/83059)
Supplement: Multimedia Appendix 1 [file diabetes-v11-e83059-s001.doc]

Multimedia Appendix 1. Search Strategy

**PubMed:**

("digital twin"[Title/Abstract] OR "digital twin"[Text Word] OR "virtual twin"[Title/Abstract] OR "virtual twin"[Text Word] OR "digital patient"[Title/Abstract] OR "digital patient"[Text Word] OR "virtual patient"[Title/Abstract] OR "virtual patient"[Text Word] OR "digital patient model"[Title/Abstract] OR "digital patient model"[Text Word] OR "virtual patient model"[Title/Abstract] OR "virtual patient model"[Text Word] OR "in silico model"[Title/Abstract] OR "in silico model"[Text Word] OR "simulation model"[Title/Abstract] OR "simulation model"[Text Word] OR "cyber-physical system"[Title/Abstract] OR "cyber-physical system"[Text Word]) AND ("personalized medicine"[Title/Abstract] OR "personalized medicine"[Text Word] OR "precision medicine"[Title/Abstract] OR "precision medicine"[Text Word] OR "individualized medicine"[Title/Abstract] OR "individualized medicine"[Text Word] OR "targeted therapy"[Title/Abstract] OR "targeted therapy"[Text Word] OR "patient-specific treatment"[Title/Abstract] OR "patient-specific treatment"[Text Word] OR "personalized"[Title/Abstract] OR "personalized"[Text Word] OR "precision"[Title/Abstract] OR "precision"[Text Word] OR "medicine"[Title/Abstract] OR "medicine"[Text Word]) AND ("diabetes*"[Title/Abstract] OR "diabetes*"[Text Word] OR "type 1 diabetes"[Title/Abstract] OR "type 1 diabetes"[Text Word] OR "type 2 diabetes"[Title/Abstract] OR "type 2 diabetes"[Text Word] OR "T1D"[Title/Abstract] OR "T1D"[Text Word] OR "T2D"[Title/Abstract] OR "T2D"[Text Word] OR "hyperglycemia"[Title/Abstract] OR "hyperglycemia"[Text Word] OR "hypoglycemia"[Title/Abstract] OR "hypoglycemia"[Text Word] OR "glycemic*"[Title/Abstract] OR "glycemic*"[Text Word])

**IEEE:**

("digital twin" in Document Title OR "digital twin" in Abstract OR "digital twin" in Index Terms OR "virtual twin" in Document Title OR "virtual twin" in Abstract OR "virtual twin" in Index Terms OR "digital patient" in Document Title OR "digital patient" in Abstract OR "digital patient" in Index Terms OR "virtual patient" in Document Title OR "virtual patient" in Abstract OR "virtual patient" in Index Terms OR "digital patient model" in Document Title OR "digital patient model" in Abstract OR "digital patient model" in Index Terms OR "virtual patient model" in Document Title OR "virtual patient model" in Abstract OR "virtual patient model" in Index Terms OR "in silico model" in Document Title OR "in silico model" in Abstract OR "in silico model" in Index Terms OR "simulation model" in Document Title OR "simulation model" in Abstract OR "simulation model" in Index Terms OR "cyber-physical system" in Document Title OR "cyber-physical system" in Abstract OR "cyber-physical system" in Index Terms) AND ("personalized medicine" in Document Title OR "personalized medicine" in Abstract OR "personalized medicine" in Index Terms OR "precision medicine" in Document Title OR "precision medicine" in Abstract OR "precision medicine" in Index Terms OR "individualized medicine" in Document Title OR "individualized medicine" in Abstract OR "individualized medicine" in Index Terms OR "targeted therapy" in Document Title OR "targeted therapy" in Abstract OR "targeted therapy" in Index Terms OR "patient-specific treatment" in Document Title OR "patient-specific treatment" in Abstract OR "patient-specific treatment" in Index Terms OR "personalized" in Document Title OR "personalized" in Abstract OR "personalized" in Index Terms OR "precision" in Document Title OR "precision" in Abstract OR "precision" in Index Terms OR "medicine" in Document Title OR "medicine" in Abstract OR "medicine" in Index Terms) AND ("diabetes*" in Document Title OR "diabetes*" in Abstract OR "diabetes*" in Index Terms OR "type 1 diabetes" in Document Title OR "type 1 diabetes" in Abstract OR "type 1 diabetes" in Index Terms OR "type 2 diabetes" in Document Title OR "type 2 diabetes" in Abstract OR "type 2 diabetes" in Index Terms OR "T1D" in Document Title OR "T1D" in Abstract OR "T1D" in Index Terms OR "T2D" in Document Title OR "T2D" in Abstract OR "T2D" in Index Terms OR "hyperglycemia" in Document Title OR "hyperglycemia" in Abstract OR "hyperglycemia" in Index Terms OR "hypoglycemia" in Document Title OR "hypoglycemia" in Abstract OR "hypoglycemia" in Index Terms OR "glycemic*" in Document Title OR "glycemic*" in Abstract OR "glycemic*" in Index Terms)

**Scopus:**

(TITLE("digital twin") OR ABS("digital twin") OR TITLE("virtual twin") OR ABS("virtual twin") OR TITLE("digital patient") OR ABS("digital patient") OR TITLE("virtual patient") OR ABS("virtual patient") OR TITLE("digital patient model") OR ABS("digital patient model") OR TITLE("virtual patient model") OR ABS("virtual patient model") OR TITLE("in silico model") OR ABS("in silico model") OR TITLE("simulation model") OR ABS("simulation model") OR TITLE("cyber-physical system") OR ABS("cyber-physical system")) AND (TITLE("personalized medicine") OR ABS("personalized medicine") OR TITLE("precision medicine") OR ABS("precision medicine") OR TITLE("individualized medicine") OR ABS("individualized medicine") OR TITLE("targeted therapy") OR ABS("targeted therapy") OR TITLE("patient-specific treatment") OR ABS("patient-specific treatment") OR TITLE("personalized") OR ABS("personalized") OR TITLE("precision") OR ABS("precision") OR TITLE("medicine") OR ABS("medicine")) AND (TITLE("diabetes*") OR ABS("diabetes*") OR TITLE("type 1 diabetes") OR ABS("type 1 diabetes") OR TITLE("type 2 diabetes") OR ABS("type 2 diabetes") OR TITLE("T1D") OR ABS("T1D") OR TITLE("T2D") OR ABS("T2D") OR TITLE("hyperglycemia") OR ABS("hyperglycemia") OR TITLE("hypoglycemia") OR ABS("hypoglycemia") OR TITLE("glycemic*") OR ABS("glycemic*"))

**WOS:**

(TS=("digital twin") OR TI=("digital twin") OR KP=("digital twin") OR TS=("virtual twin") OR TI=("virtual twin") OR KP=("virtual twin") OR TS=("digital patient") OR TI=("digital patient") OR KP=("digital patient") OR TS=("virtual patient") OR TI=("virtual patient") OR KP=("virtual patient") OR TS=("digital patient model") OR TI=("digital patient model") OR KP=("digital patient model") OR TS=("virtual patient model") OR TI=("virtual patient model") OR KP=("virtual patient model") OR TS=("in silico model") OR TI=("in silico model") OR KP=("in silico model") OR TS=("simulation model") OR TI=("simulation model") OR KP=("simulation model") OR TS=("cyber-physical system") OR TI=("cyber-physical system") OR KP=("cyber-physical system")) AND (TS=("personalized medicine") OR TI=("personalized medicine") OR KP=("personalized medicine") OR TS=("precision medicine") OR TI=("precision medicine") OR KP=("precision medicine") OR TS=("individualized medicine") OR TI=("individualized medicine") OR KP=("individualized medicine") OR TS=("targeted therapy") OR TI=("targeted therapy") OR KP=("targeted therapy") OR TS=("patient-specific treatment") OR TI=("patient-specific treatment") OR KP=("patient-specific treatment") OR TS=("personalized") OR TI=("personalized") OR KP=("personalized") OR TS=("precision") OR TI=("precision") OR KP=("precision") OR TS=("medicine") OR TI=("medicine") OR KP=("medicine")) AND (TS=("diabetes*") OR TI=("diabetes*") OR KP=("diabetes*") OR TS=("type 1 diabetes") OR TI=("type 1 diabetes") OR KP=("type 1 diabetes") OR TS=("type 2 diabetes") OR TI=("type 2 diabetes") OR KP=("type 2 diabetes") OR TS=("T1D") OR TI=("T1D") OR KP=("T1D") OR TS=("T2D") OR TI=("T2D") OR KP=("T2D") OR TS=("hyperglycemia") OR TI=("hyperglycemia") OR KP=("hyperglycemia") OR TS=("hypoglycemia") OR TI=("hypoglycemia") OR KP=("hypoglycemia") OR TS=("glycemic*") OR TI=("glycemic*") OR KP=("glycemic*"))
