# Supplementary material for: Digital Twin Applications in Diabetes Management: Scoping Review
Source: JMIR Diabetes. 2026 Jun 18;11:e83059. doi: 10.2196/83059 (PMC13277824; doi:10.2196/83059)
Supplement: Multimedia Appendix 2 [file diabetes-v11-e83059-s002.docx]

**Multimedia Appendix 2. Full-Text Articles Excluded After Eligibility Assessment**

| ID | Title | Year | DOI | Final Inclusion Decision | Exclude Reason |
| --- | --- | --- | --- | --- | --- |
| 1 | Leveraging artificial intelligence and machine learning to accelerate discovery of disease-modifying therapies in type 1 diabetes | 2025 | 10.1007/s00125-024-06339-6 | Exclude | Publication type not eligible (review article) |
| 2 | Digital Twins in Type 1 Diabetes: A Systematic Review | 2024 | 10.1177/19322968241262112 | Exclude | Publication type not eligible (review article) |
| 3 | Digital twins and artificial intelligence in metabolic disease research | 2024 | 10.1016/j.tem.2024.04.019 | Exclude | Publication type not eligible (review article) |
| 4 | Personalized Hybrid Closed-Loop Therapy Using a Digital Twin in Patients with Type 1 Diabetes: At-Home Data | 2020 | 10.2337/db20-1006-P | Exclude | Full text not accessible |
| 5 | Digital twins for telemedicine and personalized medicine | 2024 | 10.1016/B978-0-443-28884-5.00014-2 | Exclude | Publication type not eligible (review article) |
| 6 | Real-time intelligent application for lifestyle and mind state monitoring and simulation using digital twin, artificial intelligence and IoT | 2022 | Not available | Exclude | FQ2 = No (not focused on diabetes) |
| 7 | Study on using digital twin technology for monitoring patients remotely | 2025 | 10.1117/12.3060454 | Exclude | Full text not accessible |
| 8 | Digital twins for nutrition | 2022 | 10.1016/B978-0-32-399163-6.00020-2 | Exclude | Publication type not eligible (book chapter / non-original research) |
| 9 | Estimating a Personalized Basal Insulin Dose from Short-Term Closed-Loop Data in Type 2 Diabetes | 2022 | 10.1109/CDC51059.2022.9992960 | Exclude | FQ1 = No / did not meet digital twin eligibility criteria |
| 10 | Deep digital phenotyping in type 1 diabetes: The reinvention of epidemiological research | 2021 | 10.1016/j.mmm.2021.04.005 | Exclude | Not in English |
| 11 | The future of precision diabetes: Digital twin | 2024 | 10.1016/B978-0-323-98808-7.00021-7 | Exclude | Publication type not eligible (book chapter / non-original research) |
